# Insights into the Survival Capabilities of *Cryomyces antarcticus* Hydrated Colonies after Exposure to Fe Particle Radiation

**DOI:** 10.3390/jof7070495

**Published:** 2021-06-22

**Authors:** Claudia Pacelli, Cassaro Alessia, Loke M. Siong, Aureli Lorenzo, Ralf Moeller, Akira Fujimori, Shuryak Igor, Onofri Silvano

**Affiliations:** 1 Italian Space Agency, 00133 Rome, Italy; claudia.pacelli@asi.it; 2 Department of Ecological and Biological Sciences, University of Tuscia, 01100 Viterbo, Italy; lorenzo.aureli@unitus.it (A.L.); onofri@unitus.it (O.S.); 3 Ludwig Maximilian University of Munich, 80336 Munich, Germany; lokemunsiong@hotmail.com; 4 Radiation Biology Department, Aerospace Microbiology, German Aerospace Center (DLR e.V.), Institute of Aerospace Medicine, 51147 Cologne (Köln), Germany; ralf.moeller@dlr.de; 5 Department of Natural Sciences, University of Applied Sciences Bonn-Rhein-Sieg (BRSU), 53359 Rheinbach, Germany; 6 Molecular and Cellular Radiation Biology Group, Department of Basic Medical Sciences for Radiation Damages, NIRS/QST, Chiba 263-8555, Japan; fujimori.akira@qst.go.jp; 7 Department of Radiation Oncology, Center for Radiological Research, Columbia University Irving Medical Center, New York, NY 10032, USA; is144@cumc.columbia.edu

**Keywords:** radiation, melanin, DNA, radioresistance, metabolically active cells

## Abstract

The modern concept of the evolution of Mars assumes that life could potentially have originated on the planet Mars, possibly during the end of the late heavy bombardment, and could then be transferred to other planets. Since then, physical and chemical conditions on Mars changed and now strongly limit the presence of terrestrial-like life forms. These adverse conditions include scarcity of liquid water (although brine solutions may exist), low temperature and atmospheric pressure, and cosmic radiation. Ionizing radiation is very important among these life-constraining factors because it damages DNA and other cellular components, particularly in liquid conditions where radiation-induced reactive oxidants diffuse freely. Here, we investigated the impact of high doses (up to 2 kGy) of densely-ionizing (197.6 keV/µm), space-relevant iron ions (corresponding on the irradiation that reach the uppermost layer of the Mars subsurface) on the survival of an extremophilic terrestrial organism—*Cryomyces antarcticus*—in liquid medium and under atmospheric conditions, through different techniques. Results showed that it survived in a metabolically active state when subjected to high doses of Fe ions and was able to repair eventual DNA damages. It implies that some terrestrial life forms can withstand prolonged exposure to space-relevant ion radiation.

## 1. Introduction

Outer space and the surface of most celestial bodies are subjected to high fluxes of ionizing radiation, which constitute the major damaging factor in space. The radiation environment consists of high-energy photons (such as gamma rays and X-rays) and particles. Galactic cosmic rays (GCRs) are high-energy charged particles that originate beyond the solar system. They consist of 98% nuclei and 2% electrons that markedly differ in their relative abundance; a total of 85% of the nuclei component is represented by protons, 12% by alpha particles, and 1.5% by heavier nuclei [1]. Although they represent only about 18 × 10^−3^% of the GCRs spectrum [2], Fe ions are the most significant components of GCRs when the particle flux is weighted according to the energy deposition [3,4]. They are harmful for any form of terrestrial life since they can interact either directly or indirectly with all relevant biomolecules, causing dense ionization along the trajectory of the particles and secondary ionizations of various energies that can diffuse in multiple directions and to varying distances from the particle trajectory. Shielding from this kind of radiation is challenging and poses one of the principal difficulties in understanding its effects on any (terrestrial) biological organisms.

Indeed, habitability in space and on the Martian surface is highly dependent on the protection from UV and ionizing radiation, such as heavy ions [5,6,7]. Differently from Earth, Mars does not have a magnetic field to deflect energetically charged particles, including GCRs and solar energetic particles (SEP). These particles penetrate the thin Martian atmosphere and react with the regolith, the dust deposit covering solid rocks, creating an oxidizing environment on the Martian surface.

The highly radiative environment on Mars has implications for life-detection missions, since hypothetical life-forms or their remains may be damaged by ionizing radiation field [8]; this could be one of the possible explanations of the for the failure of the Curiosity rover to detect any trace of life.

Indeed, the radiation dose received by Mars is quantified by theoretical calculation and determined by the data from the Curiosity rover, which is about 0.05–0.06 or 0.076 Gy/year, respectively [8,9,10].

While the UV photons are limited in the upper layer of Martian sub-surface, as they are absorbed by rocks [9,11], GCRs have the capability to penetrate to 1–2 m in the sub-surface in spite of chemical and physics features of rocks or ice content [9]. Besides, UV flux depends on function of latitude and some molecular targets (e.g., amino acids) could be easily destroyed when embedded in highly UV-penetrated materials [12].

In addition, it was demonstrated that 1–2 mm of Mars simulant regolith layer is able to shield biological samples against UV flux [13].

On the contrary, damages provoked by ionizing irradiation are considered as one of the most dangerous. As reported in [14], an increased reduction of amplicons in a 2000 bp amplification occurs in fungal samples exposed to different doses of ionizing radiation.

At a depth of more than 2 m, it is more plausible that a hypothetical life form has been able to adapt, since the environment is partially shielded from ionizing radiation. Potentially microbial life on Mars could have persisted in protected niches in the Martian sub-surface in dried or in freezing conditions. The estimated survival duration for cryoconserved microorganisms could be about 3.3 million years at 2 m depth [5].

However, the presence of hygroscopic salts on Mars’s surface creates a transient liquid film that could be important for a putative life-form on Mars, since water is essential for life as we know it [15]. In fact, perchlorates are helpful for the presence of liquid water, but the biochemical adaptation of life in brine should be taken into account. A plausible hypothesis could be represented by the idea that deliquescence provoked by salts concentration could sustain hypothetical active microbial life under extremely radiative and dry conditions, such as those that occur in hyper-arid Atacama Desert [16].

It has been demonstrated that deliquescence in specific Martian regions could be provided by:

(i) the low thermal inertia and temperature, which allow the increment of humidity level or by
(ii) movements on the Martian surface [17].

In the presence of transient liquid water, what effects does radiation have on microorganisms?

In this context, the study of the microbial survivability against radiation effects is of importance to deepen the biological mechanisms that hypothetical life forms could assume in an extraterrestrial habitat [7]. Previous studies demonstrated the survivability of cyanobacteria, bacteria, lichens, and micro-fungi after exposure to simulated space and Martian conditions [18,19,20,21,22,23].

Despite this recognizable role of radiation in life endurance, effects of space-relevant radiation on metabolically active terrestrial organisms are still not well characterized due to the difficulties of fully reproducing the space radiation environment. The capability of the Antarctic cryptoendolithic black fungus *C. antarcticus* in resisting the space-relevant radiation has already been demonstrated. Indeed, in desiccated conditions, the fungus was able to survive the radiation environment experimented in low Earth orbit (LEO), outside the International Space Station (ISS) [20,21,22,24], and high doses of ionizing radiation exposure (gamma rays up to 55 kGy, [25]). Here, for the first time, we investigated the survival capability of the fungus under increasing doses of accelerated outer-space-relevant Fe ions radiation in hydrated (metabolically active) conditions. Indeed, in the frame of STARLIFE irradiation campaign [26], fungal colonies were irradiated in liquid medium with accelerated Fe ions doses (up to 2 kGy) at the HIMAC (Heavy Ion Medical Accelerator in Chiba) facility at the National Institute of Radiological Science (NIRS) in Chiba, Japan. Survival, metabolic activity recovery, and DNA damages were investigated.

## 2. Materials and Methods

### 2.1. Test Organism

The test organism is the black fungus *C. antarcticus* MNA-CCFEE 515, isolated by R. Ocampo-Friedmann from sandstone collected at Linnaeus Terrace in McMurdo Dry Valleys (Southern Victoria Land, Antarctica) by H. Vishniac during the Antarctic expedition of 1980–1981 [27]. This fungus was isolated through a culture-dependent approach on Malt Extract Agar (MEA) Petri dishes. This microcolonial fungus is widely used in astrobiological-related studies due its tolerance to various stressors. It is a cryophilic organism, with a growth optimum below 15 °C, which lives dwelling inside the porous surface of sandstone as refuge against the harsh Antarctica environment. A detailed description of *C. antarcticus* morphology and adaptation is reported in [28].

### 2.2. Samples Preparation and Performed Analyses

The fungal samples were grown at 15 °C for 3 months and then transferred into 200 µl of malt extract (ME) liquid medium for the irradiation (fungal concentration 1000 CFU/ml). The irradiation was performed using accelerated Fe ions (Fe^26+,^ Energy: 418.3 MeV/n, LET in water: 197.6 keV/µm; range in water: 74.4 mm) doses (up to 2 kGy), as reported in Table 1. The survival of *C. antarticus* was quantified by the CFU number on cultivation test with 5 technical replicates. The integrity of cell membranes was investigated by Propidium MonoAzide (PMA) assay and followed by quantitative PCR (qPCR) according to the protocol reported in [14]. The metabolic activity was evaluated using MTT (3-(4,5-dimethylthiazol-2-yl)-2,5-diphenyltetrazolium bromide) through a spectrophotometric assay. Detailed procedures are reported in Supplementary Materials. Lastly, the intactness of DNA regions (ITS-LSU) was examined using single gene amplifications, RAPD fingerprinting, and quantitative PCR. Conditions for DNA amplifications are reported in Supplementary Materials.

Based on surviving colony counts, mathematical modelling of cell survival dose response was performed. To quantitatively model the radiation dose response for colony survival, we used the standard linear quadratic (LQ) model of radiation-induced cell death. This formalism is commonly used in radiation biology and oncology, and here, we used a variant that takes into account the inducibility of DNA repair [29,30].

The selected formalism describes the natural logarithm of the number of surviving clonogens or colony forming units (lnS) after an exposure to an acute radiation dose d by the following equation, where lnB is the number of colonies expected without radiation, α is the linear dose response component, and β is the quadratic component that quantifies the dose response “curvature”:

lnS = lnB − α × d – β × d^2(1)

Importantly, DNA repair, which affects cell survival, potentially exhibits dose-dependent inducibility. This phenomenon was not taken into consideration by the classic LQ model but was investigated in models such as the one from [31]. Here, we used a simple variant of an inducible repair model described by the following equation, where the term q × dr with adjustable parameters q and r represents repair induction:

lnS = lnB − α/(1 + q × d^r) × d − β/(1 + q × d^r) × d^2(2)

At low doses, this model (Equation (2)) exhibits behaviours similar to the classic LQ model (Equation (1)). At high doses, however, the dose response slope (the derivative dlnS/dd) is reduced and can approach zero if parameter r approaches 2. We fitted the model (Equation (2)) to the data (ln-transformed colony counts per plate) using robust nonlinear least squares methodology implemented by the nlrob function in R 3.6.2 software. To minimize the chances of finding the global optimum best-fit solution rather than a local optimum, we performed the fitting 1000 times with random initial conditions (drawn from log-normal distributions) for the adjustable parameters (lnB, α, β, q, r) and retained the best fit. All parameters (except lnB) were restricted to non-negative values, and parameter r was restricted to 0 ≤ r ≤ 2 to maintain biological plausibility. The quality of the best fit was assessed by coefficient of determination (R2), root mean squared error (RMSE), and examination of residuals for consistency with the normal distribution (by Shapiro–Wilk test and Q-Q-plot).

## 3. Results

### 3.1. Survival Assessment

#### Cultivation Test

**Figure 1:**
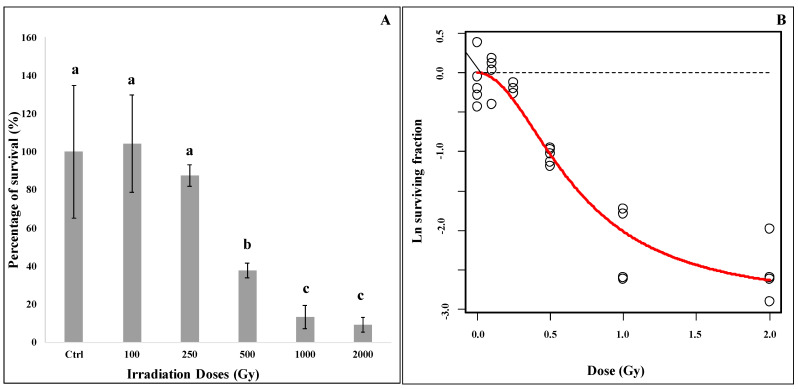
(**A**) Survival of fungal colonies in liquid media exposed to accelerated Fe ions. (**B**) Mathematical model of cell survival dose response. The same letters above bars indicate that the values are not statistically significant according to the *t* test (*p* ≤ 0.05).

As showed in Figure 1A, *C. antarcticus* retained the colony-forming ability after radiation treatments. A gradual increase of mortality was recorded with increasing radiation doses, but a considerable percentage of survivors were observed even after 1000 Gy and 2000 Gy irradiation (13% and 9%, respectively, Figure 1A). Survival counts were fitted by a dose-response curve using robust regression, as shown in Figure 1B. Visual inspection suggests decent fit quality for the dose-response model of radiation-induced cell death (described in Supplementary Materials), and this is supported by the high R2 (0.92) and relatively low RMSE (0.29 on ln scale) values. The best-fit value of the linear dose-response component (parameter α) was not significantly different from zero. The other parameters had the following best-fit values and standard errors (SE): ln-transformed background colony count (lnB) = 7.69, SE = 0.12; quadratic dose-response component (β) = 6.42, SE = 3.23 kGy-2; repair induction parameter (q) = 2.19, SE = 1.66 kGy-2; repair induction dose-dependence power (r) = 2.0, SE = 0.38. The minimum robustness weight in the regression was 0.60, and model residuals were consistent with the normal distribution (Shapiro–Wilk *p*-value = 0.65).

### 3.2. Metabolic Activity Assessment

**Figure 2:**
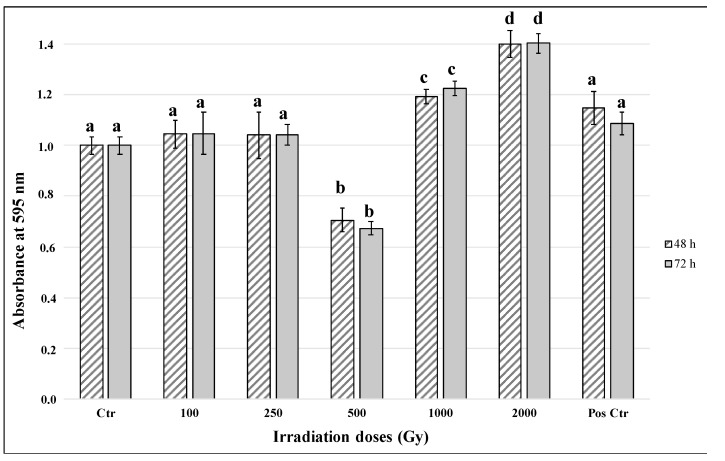
Metabolic activity of fungal cells after 48 h and 72 h rehydration. Striped bars indicate 48 h rehydration; grey bars indicate 72 h rehydration. Pos Ctr = DNA of *C. antarcticus* colony growth in physiological conditions. The same letters above bars indicate that the values are not statistically significant according to the *t* test (*p* ≤ 0.05).

No difference in the overall metabolic activity after 48 and 72 h-rehydration was recorded in irradiated fungal colonies compared with related controls (Figure 2). The fluorescent signal exhibited higher values with increasing irradiation doses. However, there was an unexpected decrease of optical density (O.D.) value in the samples that were treated with 500 Gy irradiation. Positive control samples that were kept on agar plates with sufficient required nutrient and under optimal growth conditions have shown to have higher metabolic activity than the control (Ctr, in dried conditions) sample. On the other side, samples that received irradiation dose treatments displayed high O.D. value compared to positive control samples. This phenomenon may indicate that the metabolic activity is higher in irradiated cells due to the damage repair mechanism activated upon irradiation.

### 3.3. Cell Membranes Integrity

**Figure 3:**
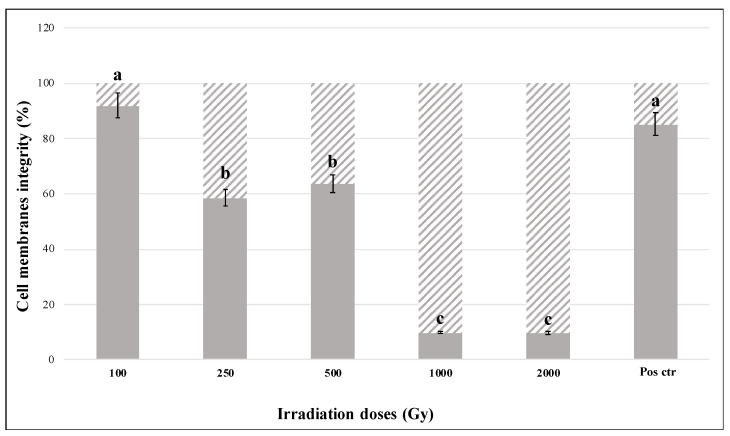
Percentage of intact (grey bars) and damaged cell-membranes (lined bars) measured with PMA assay coupled with qPCR of *C. antarcticus* exposed to accelerated Fe ions. Pos Ctr = DNA of *C. antarcticus* colony growth in physiological conditions. The same letters above bars indicate that the values are not statistically significant according to the t test (*p* ≤ 0.05).

The integrity of cell-membrane in irradiated cells was assessed by using qPCR combined with the pre-treatment of cells with the dye PMA. This molecule penetrates cells with compromised cell-membranes and inhibits DNA amplification. Accordingly, this analysis revealed a progressive damage with the increasing of treatments; an average of 39% damaged cells were recorded at the dosed of 100, 250, 500, and 1000 Gy (Figure 3). No cells with intact cell membranes were reported at the dose of 2000 Gy, according to the survival test.

### 3.4. DNA Integrity

**Figure 4:**
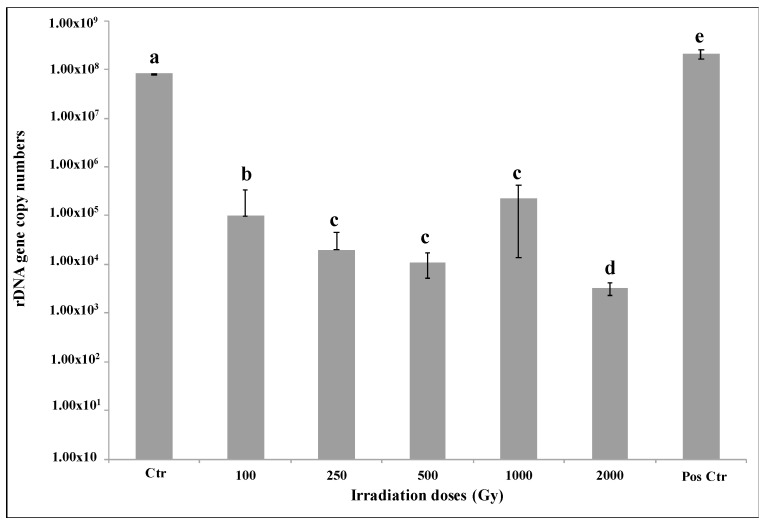
Quantitative PCR of a 939 bp (base pair) target gene (LSU) of *C. antarcticus* DNA after exposure to Fe ions irradiation. Pos Ctr = DNA of *C. antarcticus* colony growth in physiological conditions. Statistical analysis as in Figure 1.

Amplicons were obtained both for ITS and LSU regions (fragments length of 700 bp, 1600 bp and 2000 bp) of *C. antarcticus* genome after irradiation treatments. DNA amplification revealed a good DNA integrity without any differences despite gene length and treatments (Figure S1A–C). The overall RAPD profiles were shown to be preserved (Figure S2). The quantification of LSU gene fragment amplification in Figure 4 shows that an average of 15,000 DNA copies were obtained from the sample treated with doses up to 1000 Gy and approximately 1500 DNA copies from the sample treated with 2000 Gy dose. The tests highlighted a common trend for all the samples. Surprisingly, a high number of DNA copies were reported for 2000 Gy sample, although low survival rate was observed.

## 4. Discussion

In space, microorganisms have to cope with an interplay of various adverse environmental factors [32]. Besides high vacuum and extreme temperatures, they are exposed to a complex radiation environment. Assuming no shielding, the flux of cosmic ray iron (Z = 26) with energies between 100 and 1000 MeV/nucleon is approximately 4 nuclei/cm^2^ day. The ultimate limit for survival of spores in space (not on Martian surface, where the limits of life are established through a multitude of factors) may be set by the heavy ions from cosmic radiation. An approximate estimation for survival could be given on the basis of known data on the radio-resistance of terrestrial organisms.

However, it must be considered that physical conditions can significantly change the amount of radiation damage in cells at the same dose. It is known that, in aqueous environments, most of radiation damages are caused by indirect effects, such as the formation of highly reactive oxygen species like free radicals OH, O_2_H, etc. [33,34]. In the present experiment, fungal cells were exposed to accelerated Fe ions within liquid medium. However, given the linear energy transfer (LET) and range values of incident ions, no significant shielding was made against radiation independently of the position of the fungal cells during exposure.

In the optic of the search for life on Mars, such conditions allowed assessment of the effects of part of the radiation environment occurring on the surface and in the uppermost layer of Martian subsurface, where microorganisms would be directly exposed to incident CRs.

The radiolysis of water molecules in the solution by high LET Fe ions may have led to an increase of free radicals and molecular products in the environment surrounding the cells [35]. A correlation between the LET and the radical and molecular yields in water exists. In this respect, it was shown that desiccated microbial cells survive higher irradiation doses than cells in suspensions [36]. Dormant cells can accumulate damage at high levels, whereas metabolically active cells could be able to mitigate some damage [37]. However, even if cells in a desiccated state show a greater resistance to radiation exposure and could protect against any residual radiation-induced formation of reactive oxygen species (ROS) [38], the additional damaging consequences of dehydration [39] should be taken into account. On the other hand, previous work demonstrated that radiation and desiccation resistance is due to the ability to regenerate intact chromosomes from scattered fragments [40,41].

Here, in the framework of STARLIFE project, hydrated fungal samples were irradiated with iron ions for which energy (~500 MeV) represent the flux of GCRs near the Martian surface; thus, this could be considered as representative not only of Fe particles but also of most of cosmic rays making up the space and Martian radiation environments.

For the first time, our experiment evaluated the effects of high LET particles exposure on *C. antarcticus* cells in metabolically active conditions. Our results are consistent with the previously observed high resistance of the fungus to different kinds of ionizing radiation [25,42,43] and to other factors encountered in space aboard the ISS [22]. Even if the number of alive cells decreased with the increase of radiation doses, robust fungal survival was reported still at the higher doses (1000 and 2000 Gy, Figure 1A). A similar decrease of survival was observed for *Halobacterium salinarum* NRC-1 when irradiated with Fe ions up to 2 kGy [44]. The mathematical model (Figure 1B) used to describe the dose-response behavior of fungal cells is characterized by an initially shallow inactivation response (shoulder) at low doses, where the majority of cells survive, followed by a tail at higher doses. The tail, where most of the cells are dead but a few survive even at high doses, can indicate a distinct subpopulation within the fungal population that is able to survive, maybe due to the activation of DNA damage-repair mechanisms. Future researches focusing on re-irradiation of this resistant subpopulation should be performed for clarifying if fungal cells can evolve a mechanism of radio resistance. The activation of some resistance mechanisms is confirmed by the results of metabolic activity assay. Figure 2 shows an increased metabolic activity at the highest doses, probably due to the activation of an intense cell-repair mechanism. Accordingly, to the survival and metabolic activity results, the analysis on cell membrane integrity showed a reduction in the number of cells with intact membranes (and therefore presumably alive) at the dose of 500 Gy (around 60%, Figure 3); no relevant differences were detected in control samples. Moreover, the fact that around 10% and 6% of cells have intact cell membranes at the dose of 1000 and 2000 Gy (Figure 3) confirmed the survival reported from the cultivation tests and the hypothesis of the presence of radioresistant cell subpopulations. In addition, DNA damages were tested through PCR and qPCR gene amplification. In general, PCR single gene amplifications (Figure S1) were successful even at the highest dose and longest gene sequences tested; in the same way, RAPD amplification results showed a good preservation of the fingerprinting profiles (Figure S2). Based on the principle that certain DNA lesions terminate the correct course of any polymerase on the template and any nucleic acids damage may be measured as a decrease in amplification of the fragment of interest [45], qPCR analyses were performed. Our results showed that DNA amplification was not impaired by extreme radiation even at the dose of 2000 Gy (Figure 4), which reported more than 3000 copies of amplified DNA. A good amplification was detected for control samples (Figure 4). As previously reported in [44], the exposure of different bacteria strains to heavy ions reported only a few lesions in DNA, if referring to iron ions up to 1 kGy compared with the irradiation with Ar ions. This could be explained by the fact that DNA damage induced by ionizing radiation is localized [46]. To conclude, the obtained results revealed that *C. antarcticus* could be considered as a suitable candidate to search out planets beyond Earth, especially Mars, that are characterized by the presence of transient liquid water on the surface and a high-radiation environment, also with a metabolically active state. However, the mechanisms involved in the fungal radiation resistance in the presence of a thin water layer should be further investigated through -omics approaches. In addition, a further investigation about the stability of fungal biomolecules (biomarkers) after irradiation exposure could be required. The ongoing and future in situ life-detection missions on planetary bodies of our solar system (e.g., Mars) should drill the surface [47,48] in order to detect signs of past or present life. Assessing the biomarkers preservation under a highly radiative environment is of outmost importance to support these missions and to estimate the stability/degradation rates of biological molecules [49]. Besides, the investigation of the techniques could be used to evaluate the hypothetical biomolecules damages and is a test bed for the future Mars samples return (MSR) on Earth [50].

## Figures and Tables

**Table 1:** Accelerated Fe ion radiation doses applied for each sample.

Samples ^a^	Applied/ Received Dose (Gy)
Control	0
Irradiated colonies	100
Irradiated colonies	250
Irradiated colonies	500
Irradiated colonies	1000
Irradiated colonies	2000
Positive control ^b^	0

^a^ metabolically active fungal cells irradiated in liquid media. ^b^ metabolically active fungal cells maintained in optimal growth conditions.

## Data Availability

Not applicable.

## Supplementary Materials

The following are available online at https://www.mdpi.com/article/10.3390/jof7070495/s1, Material and methods; PCR and RAPD results; Figure S1 (PCR amplification), Figure S2 (RAPD amplification).
