# Supplementary material for: Insights into the Survival Capabilities of Cryomyces antarcticus Hydrated Colonies after Exposure to Fe Particle Radiation
Source: J Fungi (Basel). 2021 Jun 22;7(7):495. doi: 10.3390/jof7070495 (PMC8304246; doi:10.3390/jof7070495)
Supplement: Supplementary file 1 [file jof-07-00495-s001.zip › jof-1235620-supplementary.pdf]

Journal of *Fungi*

Article

Article

Insights into the Survival Capabilities of *Cryomyces antarcticus* Hydrated Colonies after Exposure to Fe Particle Radiation

Pacelli Claudia [1,2], Cassaro Alessia [2,*], Loke M. Siong [3], Aureli Lorenzo [2], Moeller Ralf [4,5], Fujimori Akira [6], Shuryak Igor [7] and Onofri Silvano [2]

[1] Italian Space Agency, 00133 Rome, Italy; claudia.pacelli@asi.it
[2] Department of Ecological and Biological Sciences, University of Tuscia, 01100 Viterbo, Italy; lorenzo.aureli@unitus.it (A.L.); onofri@unitus.it (O.S.)
[3] Ludwig Maximilian University of Munich, 80336 Munich, Germany; lokemunsiong@hotmail.com
[4] Radiation Biology Department, Aerospace Microbiology, German Aerospace Center (DLR e.V.), Institute of Aerospace Medicine, 51147 Cologne (Köln), Germany; ralf.moeller@dlr.de
[5] Department of Natural Sciences, University of Applied Sciences Bonn-Rhein-Sieg (BRSU), 53359 Rheinbach, Germany
[6] Molecular and Cellular Radiation Biology Group, Department of Basic Medical Sciences for Radiation Damages, NIRS/QST, Chiba 263-8555, Japan; fujimori.akira@qst.go.jp
[7] Department of Radiation Oncology, Center for Radiological Research, Columbia University Irving Medical Center, New York, NY 10032, USA; is144@cumc.columbia.edu
* Correspondence: cassaro@unitus.it; Tel.: +39-0761357138

Citation: Claudia, P.; Alessia, C.; Siong, L.M.; Lorenzo, A.; Ralf, M.; Akira, F.; Igor, S.; Silvano, O. Insights into the Survival Capabilities of *Cryomyces antarcticus* Hydrated Colonies after Exposure to Fe Particle Radiation. *J. Fungi* **2021**, *7*, 495. https://doi.org/10.3390/jof7070495

Academic Editors: Macit Ilkit and Sybren de Hoog

Received: 10 May 2021
Accepted: 15 June 2021
Published: 22 June 2021

Publisher's Note: MDPI stays neutral with regard to jurisdictional claims in published maps and institutional affiliations.

Copyright: © 2021 by the author. Licensee MDPI, Basel, Switzerland. This article is an open access article distributed under the terms and conditions of the Creative Commons Attribution (CC BY) license (http://creativecommons.org/licenses/by/4.0/).

Material and methods

Survival assessment

Cultivation test

Cultivation test was performed to determine the survival of fungal colonies after exposure to increasing doses of accelerated Fe ions. The colonies were diluted to a final concentration of 50000 CFU/ml, and 0.1 ml (5000 CFUs), after counting cells with light microscope. For each sample 100 µl were spread on MEA Petri dishes in quintuplicate. Samples were incubated at 15 °C for 3 months and the grown colonies were counted. Non-irradiated samples were kept in laboratory under room temperature and used as controls.

Metabolic activity assessment by MTT (3-(4,5-dimethylthiazol-2-yl)-2,5-diphenyltetrazolium bromide) assay

To perform MTT assay, a solution with 0.5 mg/ml of MTT (Thermo Fisher Scientific, Massachusetts, USA) salt in Phosphate-Buffered Saline (PBS) was added to a 100 µl of cell suspensions (3.5×105 cells/ml) to react with NAD(P)H-dependent cellular oxidoreductase of the viable cells. After incubation in a 96-well microplates in the dark at room temperature for 48 and 72 h, MTT solution was removed with a multi-channel pipette and 100 µl of DMSO (DiMethyl SulfOxide) was added. The absorbance was read at 595 nm, and the average absorbance of wells containing only MTT was subtracted from the others. The obtained MTT values were normalized by the number of cells per well, and these values were again normalized with the laboratory controls.

Membrane damage assessment

Cell membranes integrity

Propidium MonoAzide (PMA) assay was performed to evaluate cell membranes integrity, using a quantitative PCR (qPCR) to highlight the percentage of intact or damaged cells. 5 µl of a PMA solution was added to samples and incubated in the dark for 1 h with constant shaking. Then, samples were incubated in ice and exposed to a halogen lamp for 10 min. PMA can selectively penetrate cells with damaged membranes and cross-link to DNA under exposure to light, thereby preventing PCR. DNA extraction and purification were performed on PMA treated and untreated aliquots from each sample. DNAs were quantified and normalized at the same concentration (2 ng/ml) using the Qubit dsDNA HS Assay Kit (Thermo Fisher Scientific, Massachusetts, USA) and qPCR was performed to quantify the number of fungal internal transcribed spacer (ITS) ribosomal DNA fragments (281 bp) in both PMA-treated and untreated samples, according to [1]. All tests were performed in triplicate.

DNA integrity assessment

DNA extraction was performed on fungal cells using NucleoSpin® Plant kit (Macherey-Nagel, Düren, Germany) following the protocol optimized for fungi [2]. Quantitation of extracted genomic DNA was performed using Qubit system and all the samples were diluted to the same concentration (1 ng/ml). Three overlapping tracts in the Internal Transcribed Spacer (ITS) regions and the Large SubUnit-coding Sequences (LSU) of the nuclear ribosomal RNA (rRNA) gene complex were amplified. The primers ITS4 (ATTTGAGCTGTTGCCGCTTCA), ITS5 (GGAAGTAAAAGTCGTAACAAGG), LR5 (TCCTGAGGGAAACTTC) and LR7 (TACTACCACCAAGATCT) were employed. PCR reactions were carried out for each sample in a solution consisting of 12.5 ml of BioMixTM (BioLine Ltd., London), 1 ml of each primer solution (5 pmol/µl) and 0.1 ng of DNA template, in a final volume of 25 ml. MyCycler Thermal Cycler (Bio-Rad Laboratories GmbH, Munich, Germany) equipped with a heated lid were used and amplification conditions are as reported in [3]. The whole genome integrity was assessed by Random Amplified Polymorphic DNA (RAPD). PCR reactions were carried out for each sample in a final solution containing 12.5 ml of BioMixTM, 5 pmol of primer $(GGA)_7$ and 0.1 ng of DNA sample, in a final volume of 25 ml. Amplifications were performed according to [3].

Quantitative assay of DNA damage by qPCR

Quantitative PCR was carried out to quantify the number of ITS fragments by using LR0R and LR5 primers. The qPCR reactions were performed in triplicate with a solution containing 7.5 µl of qPCR cocktail (iQ SYBER Green Supermix, Biorad, MI, Italy), 1 µl of each primer solution (5 pmol/µl) and 0.1 ng of DNA template in a final volume of 15 ml. The amplifications were carried out by Biorad CFX96 real time PCR detection system.

Results

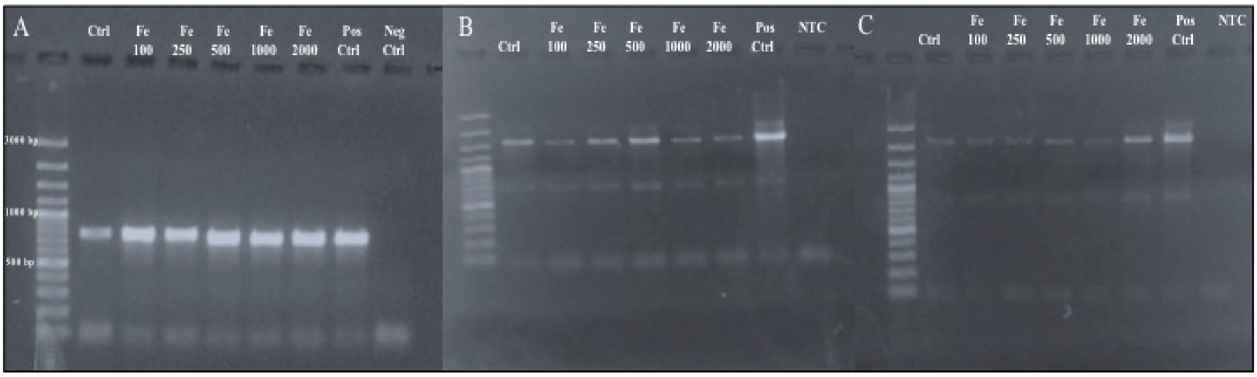

Figure S1. A) PCR amplification of the A) ITS region (700 bp), B) ITS-LSU region (1600 bp) and C) ITS-LSU region (2000 bp). Pos Ctrl: Positive Control; NTC: negative control. All irradiation treatments are expressed in Gy.

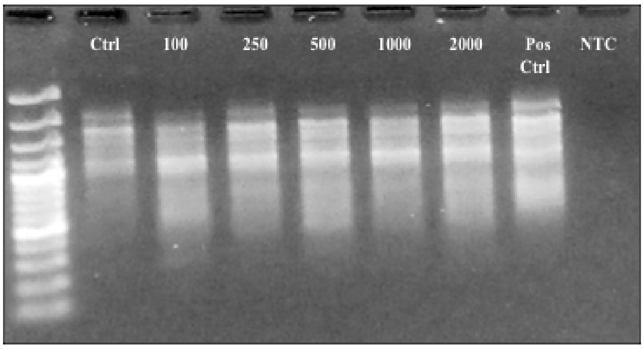

Figure S2. A) PCR amplification of the A) ITS region (700 bp), B) ITS-LSU region (1600 bp) and C) ITS-LSU region (2000 bp). Pos Ctrl: Positive Control; NTC: negative control. All irradiation treatments are expressed in Gy.

References

1. Onofri, S.; De La Torre, R.; De Vera, J.P.; Ott, S.; Zucconi, L.; Selbmann, L.; Scalzi, G.; Venkateswaran, K.J.; Rabbow, E.; Sánchez Iñigo, F.J.; et al. Survival of rock-colonizing organisms after 1.5 years in outer space. *Astrobiology* **2012**, *12*, 508–516, doi:10.1089/ast.2011.0736.
2. Selbmann, L.; De Hoog, G.S.; Mazzaglia, A.; Friedmann, E.I.; Onofri, S. *Fungi at the edge of life: cryptoendolithic black fungi from Antarctic desert*; **2005**; Vol. 51;.
3. Selbmann, L.; Isola, D.; Zucconi, L.; Onofri, S. Resistance to UV-B induced DNA damage in extreme-tolerant cryptoendolithic Antarctic fungi: Detection by PCR assays. *Fungal Biol.* **2011**, *115*, 937–944, doi:10.1016/j.funbio.2011.02.016.
